# Understanding the Relationship between Vaccine Supply Dead Space and Wasted COVID-19 Vaccine Doses

**DOI:** 10.1590/0037-8682-0353-2023

**Published:** 2023-09-22

**Authors:** Yago Marcos Pessoa-Gonçalves, Ana Lucia Gonçalves de Jesus, Chamberttan Souza Desidério, Gabrielly Amanda Minchio, Arthur de Sousa Louzada, Marcos Massao Shimano, Carlo José Freire Oliveira

**Affiliations:** 1 Universidade Federal do Triângulo Mineiro, Instituto de Ciências Biológicas e Naturais, Laboratório de Imunologia e Bioinformática, Uberaba, MG, Brasil.; 2 Prefeitura Municipal de Uberaba, Departamento de Saúde Municipal, Vigilância Epidemiológica, Uberaba, MG, Brasil.; 3 Universidade Federal do Triângulo Mineiro, Departamento de Engenharia Mecânica, Uberaba, MG, Brasil.

**Keywords:** Dead Space, Needles, Residual volume, Syringes, Vaccine Supplies

## Abstract

**Introduction:**

By July 2023, Brazil had administered approximately 540 million COVID-19 vaccine doses. This study aimed to quantify wasted doses resulting from dead space in vaccine supplies.

**Methods:**

The vaccine supply was initially weighed, filled with distilled water, and expelled to simulate administration. Weighing it again after the application determined the dead space volume. Descriptive analyses calculated the waste rate/wasted dose count.

**Results:**

The estimated total number of wasted vaccine doses using supplies with the lowest dead space was 62,097,338.

**Conclusions:**

Syringe dead space is a crucial factor in dose wastage, directly influencing the number of wasted doses.

The COVID-19 pandemic has heightened concerns regarding the quantity and consumption of vaccine supplies, particularly those associated with the administration of hypodermic-type vaccines^1^ ^-^ ^3^. Moreover, the quality of the supplied vaccines is crucial in managing health crises like the COVID-19 pandemic. Among the factors influencing the quality of these supplies, the significance of a low dead space can not be overstated^4^ ^-^ ^7^.

The International Organization for Standardization 7886-1 (ISO 7886-1), which oversees standardization in various areas including medical supplies, stipulates that hypodermic syringes with a volume less than 5 mL should have a dead space not exceeding 0.07 mL^8^ ^,^ ^9^. Consequently, pharmaceutical companies are required to include an overfill volume when filling vaccine vials to compensate for the anticipated loss due to the syringe’s dead space. A smaller dead space in the syringe results in a reduced overfill volume, thereby increasing the availability of vaccines for the production of new vaccine vials^10^ ^-^ ^12^.

The primary elements affecting the dead space volume in vaccine supplies include the syringe barrel, the area facilitating needle attachment, and the needle itself, as depicted in Figure 1A. The inclusion of a retention plug that penetrates the barrel augments the expulsion of the vaccine content, thereby effectively diminishing the dead space. The incorporation of this modified retention plug primarily distinguishes a low-dead-space (LDS) syringe from a high-dead-space (HDS) syringe, as demonstrated in Figure 1B^13^ ^-^ ^15^.

**FIGURE 1:**
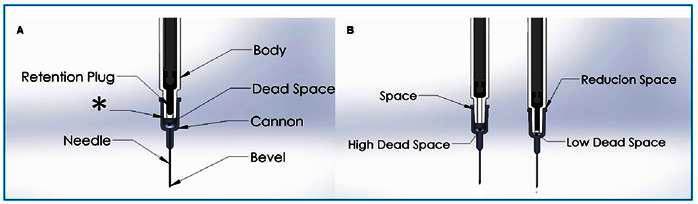
a) The illustration depicts the different components of a vaccine supply. One key finding is that the syringe cannon is the location that presents the most dead space in the supplies. In *, emphasize that the height at which the syringe and needle fit directly influences the dead space, noting that the deeper the barrel penetrates into the syringe, the smaller the dead space. **b)** The schematic drawing illustrates two types of 1 mL syringes, a high dead space syringe and a low dead space syringe. It is observable that in LDS syringes there is less dead space due to the modified retention plug and better coupling of the cannon to the syringe when compared to HDS syringes.

The direct impact of using supplies, such as syringes and needles, in conjunction with a HDS on the extraction of the total recommended doses from multidose vials for COVID-19 has been established. This was evidenced in research conducted on the Pfizer vaccine, which is pre-set to yield seven doses (0.3 mL each, culminating in a total of 2.1 mL of vaccine per vial)^6^. It is understood that the available vaccine volume per vial post-dilution is 2.25 mL, resulting in a residual volume of 0.15 mL or 0.021 mL per dose. Consequently, researchers have indicated that the extraction of these seven doses is improbable with the current vaccine supplies due to the minimal overfill and the HDS (exceeding 0.021 mL per application) of the accessible equipment^2^ ^,^ ^6^.

The CoronaVac vaccine, produced by the Butantan Institute, advises extracting ten 0.5 mL doses, yielding a total of 5 mL of vaccine per vial. Initially, the vial’s fill volume was 6.2 mL, but it was subsequently reduced to 5.7 mL. Consequently, it became impossible to extract the recommended ten doses per vial, a problem also reported by other researchers ^6^ ^,^ ^12^.

The objective of this study was to measure the dead space in various vaccine supplies utilized in Brazil and determine if certain combinations of syringes and needles result in less dead space. Furthermore, the study sought to calculate the waste rate and the number of wasted COVID-19 vaccine doses attributable to dead space.

To quantify dead space, we procured syringes and needles of varying graduations from different manufacturers, all of which are frequently used in COVID-19 vaccination procedures. The vaccine supplies utilized in this study include the SR^®^ 1 mL and 3 mL syringes, and SR® 20× 0.55 mm and 25× 0.7 mm needles (Saldanha Rodrigues, Manaus, AM, Brazil); BD PrecisionGlide™ needles in sizes 20× 0.55 mm, 25× 0.7 mm, and 25× 0.8 mm (Becton Dickinson, Curitiba, PR, Brazil); the GLOBALIX 3 mL syringe (Hunan Luzhou, Suining, China); BD Eclipse needles in sizes 25× 0.7 mm and 25× 0.8 mm (Becton Dickinson, Curitiba, PR, Brazil); the Syringe Need 3 mL and the Need 30× 0.7 mm needle (Shanghai Kindly Enterprise, Jiading-Shanghai, China); BD PLASTICK™ 1 mL syringes and SoloMed™ 3 mL syringes (Becton Dickinson, Curitiba, PR, Brazil); the Wiltex 25× 0.7 mm needle (Yangzhou Medicine Industry, Yangzhou, China); the Decarpack 3 mL syringe and 25× 0.7 mm needle (Lifelong Meditech, Manesar Gurgaon, India); the BD SoloMed™ 30× 0.7 mm needle (Becton Dickinson, Curitiba, PR, Brazil); and the Evercare 3 mL syringe and 25× 0.7 mm Evercare needle (Even Green; São Bernardo do Campo, SP, Brazil).

Each vaccine sample was weighed post-packaging removal using a calibrated ATY224 analytical electronic scale balance (Shimadzu, Kyoto, Japan) to determine the initial mass (IM). Subsequently, the vaccine supply was filled to its maximum graduation with distilled water, and the contents were expelled to mimic vaccine administration. The vaccine was then reweighed to ascertain the final mass (FM). Given that the density of distilled water approximates 1 g/cm³, the dead space volume was calculated by determining the difference between the FM and IM. This procedure was performed three times to reduce errors, and the mean and standard deviations were subsequently calculated. 

The wastage rate, determined through Equation 1, signifies the proportion of the vaccine left in the needle because of dead space and the volume of a single administered vaccine dose.

$$\text{Wastage rate}=\left(\frac{\text{Dead Space}}{\text{Volume of a single dose}}\right)*100$$ (1)

The quantity of wasted doses (Qw), as determined by Equation 2, signifies an estimated measure of COVID-19 vaccine doses wasted due to dead space.

Qw=Wastage rate *Number of doses administered (2)

Upon evaluating various combinations of vaccination supplies, namely syringe and needle, the analysis revealed that the BD PLASTICK™ 1 mL syringe paired with the Evercare 25×0.7 mm needle resulted in the least dead space (0.0433 mL). Conversely, the combination of the GLOBALIX 3 mL syringe and the BD PrecisionGlide™ 25×0.8 mm needle yielded the most dead space (0.1228 mL). These findings are detailed in Table 1.

**TABLE 1:** Descriptive analyzes of dead space in vaccine supply.

			Waste Rate (%)			
Vaccines supplies	Description	DSM ± SD (mL)	Dose 0.5 mL	Dose 0.3 mL	Dose 0.2 mL
Syringes	Syringe BD PLASTICK™ 1 mL	0.0329 ± 0.0012	6.58%	10.97%	16.45%
	Syringe BD SoloMed™ 3 mL	0.0406 ± 0.0009	8.12%	13.53%	20.30%
	Syringe SR® 3 mL	0.0408 ± 0.0028	8.16%	13.60%	20.40%
	Syringe Need 3 mL	0.0472 ± 0.0011	9.44%	15.73%	23.60%
	Syringe Descarpack 3 mL	0.0488 ± 0.0015	9.76%	16.27%	24.40%
	Syringe SR® 1 mL	0.0509 ± 0.0003	10.18%	16.97%	25.45%
	Syringe Evercare 3 mL	0.0662 ± 0.0017	13.24%	22.07%	33.10%
	Syringe GLOBALIX 3 mL	0.0688 ± 0.0096	13.76%	22.93%	34.40%
Needles	Needle Evercare 25x0.7 mm (22Gx1")	0.0104 ± 0.0045	2.08%	3.47%	5.20%
	Needle BD Eclipse 25x0.7 mm (22Gx1'')	0.0212 ± 0.0002	4.24%	7.07%	10.60%
	Needle Need 30x0.7 mm (22Gx11/4'')	0.0309 ± 0.0009	6.18%	10.30%	15.45%
	Needle BD Eclipse 25x0.8 mm (21Gx1'')	0.0349 ± 0.0014	6.98%	11.63%	17.45%
	Needle BD SoloMed™ 30x0.7 mm (22Gx1 1/4'')	0.0386 ± 0.0005	7.72%	12.87%	19.30%
	Needle SR® 20x0.55 mm	0.0410 ± 0.0012	8.20%	13.67%	20.50%
	Needle SR® 25x0.75 mm	0.0413 ± 0.0017	8.26%	13.77%	20.65%
	Needle Wiltex 25x0.7 mm (22Gx1'')	0.0429 ± 0.0015	8.58%	14.30%	21.45%
	Needle Descarpack 25x0.7 mm	0.0489 ± 0.0018	9.78%	16.30%	24.45%
	Needle BD PrecisionGlide™ 20x0.55 mm (24G x 3/4'')	0.0491 ± 0.0002	9.82%	16.37%	24.55%
	Needle BD PrecisionGlide ™ 25x0.7 mm (22Gx1'')	0.0493 ± 0.0005	9.86%	16.43%	24.65%
	Needle BD PrecisionGlide ™ 25x0.8 mm (21G x 1'')	0.0540 ± 0.0008	10.80%	18.00%	27.00%

DSM: Dead Space Mean; SD: Standard Deviation

As of July 2023, Brazil had administered a total of 540,870,616 doses of the COVID-19 vaccine. Upon analysis, using the formula for wasted doses and considering only the minimum dead space in syringes and needles identified in our study, along with the varying doses of vaccines, we estimated that the total number of vaccine doses wasted due to dead space was 62,097,338. This data is presented in Table 2.

**TABLE 2:** Number of doses administered, and number of doses wasted of Covid-19 vaccine.

Vaccine Name	Number of doses administered	Volume per dose (mL)	Number of wasted doses¹
PFIZER - COMIRNATY	200,995,589	0.3	29,023,763
ASTRAZENECA/FIOCRUZ - COVISHIELD	145,568,680	0.5	12,606,248
SINOVAC/BUTANTAN - CORONAVAC	111,936,409	0.5	9,693,694
JANSSEN - Ad26.COV2.S	30,548,685	0.5	2,645,516
PFIZER - COMIRNATY BIVALENT	25,233,957	0.3	3,643,783
PEDIATRIC - PFIZER COMIRNATY²	11,296,099	0.2	2,445,605
ASTRAZENECA - ChAdOx1-S	7,938,954	0.5	687,513
PFIZER - COMIRNATY PEDIATRIC²	2,928,264	0.2	633,969
PFIZER - COMIRNATY PEDIATRIC³	2,572,212	0.2	556,884
SINOVAC - CORONAVAC X	1,851,767	0.5	160,363

¹ Calculating wasted doses using the lowest waste rate ²Children between the ages of 5 and 11 years ³Children below 5 years of age.

Utilizing the vaccine supply combination with the smallest dead space (BD PLASTICK™ 1 mL syringe with Evercare 25×0.7 mm needle) for the 0.5 mL, 0.3 mL, and 0.2 mL doses, the waste rates are 8.66%, 14.44%, and 21.65%, respectively. Conversely, when employing the vaccine supply combination with the largest dead space (GLOBALIX 3 mL syringe with BD PrecisionGlide™ 25×0.8 mm needle) for the 0.5 mL, 0.3 mL, and 0.2 mL doses, the waste rates are 24.56%, 40.93%, and 61.40%, respectively.

Upon analyzing the combination with the lowest dead space, a total of 62,097,338 wasted doses were recorded, comprising 25,793,334 doses for 0.5 mL vaccines, 32,667,546 for 0.3 mL vaccines, and 3,636,458 for 0.02 mL vaccines. Conversely, the combination with the highest dead space resulted in an estimated total of 176,059,458 wasted doses, including 73,150,608 doses for 0.05 mL vaccines, 92,595,753 for 0.3 mL vaccines, and 10,313,097 for 0.02 mL vaccines. Therefore, the discrepancy in wasted doses between the vaccine supplies with the lowest and highest dead space was 113,962,120 doses. 

The syringes adhered to the standards delineated by ISO 7886-1^9^ ^,^ ^13^. Similar to previous studies, the dead space varied based on the supplier or the combination of syringes and needles, leading to disparate rates of waste^2^ ^,^ ^4^ ^,^ ^6^ ^,^ ^10^ ^,^ ^13^. None of the potential combinations yielded a dead space less than or equal to 0.021 mL, thereby precluding the extraction of seven doses from each Pfizer vaccine vial. Consequently, it is clear that the current vaccine supplies exhibit HDS, and the overfill in Pfizer vials is inadequate to procure the full seven doses^6^.

Conversely, numerous combinations exist for the complete extraction of 10 doses from CoronaVac. The vaccine’s overfilling permits a dead space of up to 0.07 mL per dose. As a result, combinations of syringes and needles were identified that offered a dead space equal to or less than 0.07 mL. However, it is important to acknowledge that these supplies, with reduced dead space, may not always be readily available at all locations^10^ ^-^ ^12^.

Approximately 62 million doses were wasted, as estimated, using the optimal combination of needles and syringes. The waste produced following each use of both single-dose and multidose vaccines, coupled with the inability to extract full doses from multidose vaccines, could potentially escalate public expenditures due to the necessity for additional vaccine procurement.

The disparity in waste could amount to 113,962,120 doses when comparing the utilization of supplies with LDS and HDS. This underscores the significance of procuring superior vaccine supplies with the maximum possible LDS, as it can conserve doses and minimize waste.

Overfill significantly impacts the administration of mass vaccination programs, such as the one implemented for COVID-19. In a set of vials, the vaccine volume remained consistent, yet the number of doses derived from the same volume fluctuated based on the overfill value. Consequently, the more vaccines available to generate increased doses, the less the necessity for overfilling. This reduction leads to enhanced vaccine coverage and expedites the mass vaccination process. 

A limiting factor in our study is the inability to accurately determine the specific needles and syringes used for each dose. Consequently, we can only estimate the potential number of wasted doses.

The dead space fluctuates with various needle and syringe combinations. Even with the combination yielding the least dead space in our study, the volume remained significant. Consequently, it is virtually impossible to extract the number of doses specified on multidose vials, leading to increased vaccine waste. Therefore, it is crucial to underscore the importance of using vaccines with LDS. This approach primarily aims to minimize overfill, resulting in reduced waste, an increased number of doses per vaccine volume, and enhanced efficiency during the vaccination process.
